# Honey infused with herbs: A boon to cure pathological diseases^☆^

**DOI:** 10.1016/j.heliyon.2023.e23302

**Published:** 2023-12-04

**Authors:** Suresh Kumar, Mamta Verma, Younis Ahmad Hajam, Rajesh Kumar

**Affiliations:** a Department of Biosciences, Himachal Pradesh University, Shimla, H.P., 171005, India; b Department of Life Sciences and Allied Health Sciences, Sant Baba Bhag Singh University, Jalandhar, Punjab, India

**Keywords:** Honey, herbs, therapeutic, Diseases, Infections

## Abstract

Healing with herbs has been a common practice for ages. Nowadays, various infectious diseases like malaria, flu, hepatitis B; COVID-19, etc. are commonly spreading around the world as a consequence of environmental pollution and related consequences. These diseases are not well controlled by the present drug treatment. Antibiotics are failing because of bacterial resistance. Although people believe that herbal medicines are more effective and safer. Therefore, traditional herbal remedies have been recommended for treatment purposes throughout the world. They are often used in combination, fused with honey, or alone for curing different types of ailments. Today, modern formulations of these medicines exist in the form of capsules, tablets, powders, and granules. In several traditional systems, ‘Honey’ is recommended as a natural medicine that improves several health conditions. In ‘Ayurveda’, honey is considered a most precious and miraculous product of nature and is used to treat various diseases either alone or after its infusion with herbs. It is a natural, antioxidant-rich, and highly nutritious food that is widely used as a natural sweetener without any side effects. It has antibacterial, antiviral, antifungal, and antioxidant properties. It also proves fruitful in managing/curing various disorders like colds, coughs, cancer, diabetes, wound healing, and cardiovascular disorders. Honey infused with herbs is also used to repair wounds, diabetes, lymphedema, and the prevention of chronic venomous diseases as a part of the folk medicinal system. The current article aims to analyse the medicinal efficiency of honey infused with herbs for curing/managing/treating various types of ailments.

## 1 Introduction

Honey is a sugary, viscid, thick, organic substance that's manufactured by honey bees (*Apis*s pp.). It has been used by all human civilizations forages, due to its sweet taste and therapeutic properties. Honey formation starts by gathering nectar from nectarines of blooming flowers by bees using their proboscis, which is then brought to the hive and stored inside frames. Nectar is then transformed into honey by adding enzymes and continuous fanning that cause evaporation and lead to the formation of viscous honey. The color and flavor of honey are based on the nectar that is collected by the bees [1]. Honey has been used for the purpose of nutrition as well as medicine for centuries. The first written authentication regarding the use of honey as a natural sweetener has been reported in a Sumerian tablet which dates back to 2100–2000 BCE According to archaeological records, the use of honey by humans began about 8000 years ago [2]. The use of honey for various purposes has also been documented in ancient Indian literature including Vedas and Ayurveda (about 4000 years ago). Honey is recommended as a natural medicine that improves several health conditions [3]. Due to the presence of various medicinally important compounds, honey is commonly used for the formulation of different supplements, medicines, personal care products, etc. (Table 1)

**Table 1:** Therapeutic role of honey and its constituents in the treatment of different diseases.

Bioactive Component	Medicinal Importance	Treatment of Diseases
Flavonoids	Antioxidant, anti-inflammatory	Cardiovascular diseases, cancer prevention
Phenolic Acids	Antioxidant, anti-inflammatory	Heart diseases, digestive disorders
Enzymes (e.g., Glucose Oxidase)	Antimicrobial	Wound healing, antibacterial properties
Amino Acids	Nutrient source, immune system support	Muscle recovery, overall health
Vitamins and Minerals	Nutritional support	Boosting immunity, overall health
Hydrogen Peroxide	Antiseptic	Wound healing, antibacterial properties
Defensin-1, Methylglyoxal (MGO),	Antimicrobial, anti-inflammatory	Immune system support, wound healing
Phenolic Acids (caffeic acid, *p*-coumaric acid, and ellagic acid	Antioxidant and anti-inflammatory	Scavenges free radicals, stimulates production of anti-inflammatory cytokines
Polyphenols	Nutrient source, antioxidant	Allergies, overall health support
Polyphenols	Nutrient source, immune system support	Energy, fertility support

Honey is called “Madhu” and is used as food, and medicine and also has religious features. In Indian Vedas, honey is considered “Amrut” and has its place as one of the ingredients in Panchamrita along with ghee, milk, curd, and sugar. There are certain rituals performed with honey like the ‘Madhu Abisheka’ ritual in which Madhu (honey) is poured on the deities. As a part of Hindu culture, it is commonly used in the birth ritual “Jatakarma” in which honey is given to the neonatal even before the mother's milk. According to the Ayurvedic system, honey infused with herbs is highly beneficial for weak digestion, teeth and gums, wounds, worms, and the treatment of insomnia [4].Honey is rich in antioxidants that make it effective in protecting the skin from free-radical damage and regulates enzyme activity to make the skin glow. Honey is an anti-aging solution for all skin types with hydrating properties to plump up the skin and reduce the appearance of wrinkles. It is used as an ointment for burns, wounds, and skin irritation. It is also used as first-aid dressing material by people inhabiting remote areas [5].

## 2 Urgency and reason for writing this review

The increasing prevalence of these ailments demands a critical analysis of alternative therapeutic options, especially those with the potential for safe, accessible, and effective treatment. This review aims to explore the urgency of incorporating herb-infused honey as a viable solution within contemporary healthcare practices. With its rich medicinal properties and proven efficacy in traditional remedies, there is a critical need to bridge the gap between ancient wisdom and modern scientific validation. By elucidating the mechanisms underlying the healing properties of herb-infused honey and assessing its impact on a variety of pathological conditions, we can pave the way for novel, accessible, and potentially life-changing treatments. This review intends to present a compelling case for the inclusion of this natural remedy within the mainstream medical paradigm.

### 2.1 Historical significance of herbal remedies in traditional medicine

The global use of herbal medicines for various health challenges is rapidly expanding, garnering significant public interest and acceptance across both developing and developed nations. Herbal remedies are now accessible not only in drug stores but also in food establishments. In developing countries, they serve as the primary healthcare source for a large population. India, in particular, boasts a rich traditional system of medicine, including Ayurveda, Unani, Homeopathy, and Sidha, which heavily rely on herbal treatments. The safety and efficacy of these traditional medicines have gained trust over thousands of years of use.

Natural herbal products are increasingly utilized in the development of modern drugs, dietary supplements, cosmetics, and ingredients for food and beverages. These products contain naturally occurring compounds that exhibit antiviral, antibacterial, anti-protozoal, and antioxidant properties (Table 2). The rise of drug resistance has led to a surge in the use of herbal medicine when conventional treatments fail.

**Table 2:** Therapeutic role of medicinal plants and their phyto-bioactive components against various ailments.

Medicinal Plant	Phytochemical Component	Medicinal Importance	Common Uses
Turmeric	Curcumin	Anti-inflammatory, antioxidant, antimicrobial	Arthritis, digestive disorders, wound healing
Ginger	Gingerol	Anti-nausea, anti-inflammatory, antioxidant	Nausea, digestive issues, pain relief
Garlic	Allicin	Cardiovascular health, antimicrobial, anti-inflammatory	Lowering blood pressure, boosting immunity
Echinacea	Echinacoside, Echinacein	Immune system support, anti-viral	Common cold, flu
Aloe Vera	Aloin, Aloe-emodin	Wound healing, anti-inflammatory	Skin burns, minor cuts
Lavender	Linalool, Linalyl acetate	Relaxant, anti-anxiety, anti-inflammatory	Anxiety, sleep disorders
Ginseng	Ginsenosides	Energy booster, adaptogen	Fatigue, stress
Milk Thistle	Silymarin	Liver health, antioxidant	Liver detoxification
Peppermint	Menthol	Digestive aid, anti-nausea	Indigestion, nausea
Ginkgo Biloba	Ginkgolides, Flavonoids	Cognitive function, antioxidant	Memory enhancement

The Indian herbal industry extensively employs numerous plant species, utilizing the collective therapeutic wisdom passed down through generations of physicians. While many plants have demonstrated potential antimicrobial properties in vitro, their efficacy in controlled clinical trials remains largely untested. In the face of growing antibiotic resistance, the search for alternative treatments, including herbal antibiotics, has become imperative, particularly for bacterial infections. The bioactive constituents work synergistically, optimizing therapeutic efficacy while minimizing adverse effects. This integrative approach could serve as an alternative to conventional pharmaceutical interventions. The exploration of honey infused with herbs could present a promising therapeutic intervention against the various prevalent dreadful diseases. Understanding these mechanisms can lead to the development of novel, cost-effective, and sustainable treatment modalities, addressing the unmet clinical needs of diverse patient populations. It could emphasize the necessity for collaborative research within the scientific community to fully explore the potential benefits of honey infused with herbs, suggesting that this approach could herald a new era of integrative medicine, combining the best of nature's wisdom with cutting-edge scientific advancements.

## 3 Methods

A literature search was conducted to identify recent articles illustrating the efficacy of honey in the cure of diseases. We searched for articles published from years 2005–2023 in Google Scholar. Several online databases were queried, including Web of Science, Science Direct, and PubMed. The following keywords were used individually and in combination as inclusion criteria for articles to be considered for this review: honey antioxidant, anti-inflammatory, antibacterial, anti-diabetic, apoptotic, respiratory, gastrointestinal, cardiovascular, and nervous system. A few old citations have to be mentioned wherever needed. We searched for articles in Science Direct, Elsevier, and Springer for honey, herbs, and infused formulations: therapeutic role of herbs, honey, phytoconstituents, and infusion formulations. The latest articles related to the therapeutic potency of honey, herbs, their antioxidant potency, anti-inflammatory, anticancer, and anti-diabetic were also searched in Nature Portfolio, and databases used (SCOPUS and WoS). Moreover, we also searched available treatments for various pathological diseases such as viral diseases (COVID-19), metabolic disorders (diabetes, obesity, hyperlipidemia etc.), reproductive disorders (PCOS, infertility, oligospermia, hormonal disorders), neurodegenerative diseases (Alzheimer's Parkinson disease, etc.), Hepatotoxicity, renal toxicity. Initial searches yielded nearly 200 results. The abstracts of these papers were reviewed to confirm applicability. After considering additional exclusion criteria (non-English language, and manuscripts not available as full text), approximately 110research/review papers were critically examined for compilation of present article.

## 4 Results

### 4.1 Impact of pathological diseases

Pathological diseases exert a far-reaching impact on individuals, communities, and global healthcare systems, profoundly affecting morbidity, mortality, quality of life, and socioeconomic burdens. These diseases, including infectious and chronic conditions, contribute significantly to the global burden of illness, leading to long-term health complications, reduced life expectancy, and heightened rates of disability and impairment. Consequently, individuals grappling with these conditions experience a decline in their overall quality of life, grappling with symptoms such as pain, fatigue, cognitive impairment, and physical limitations, which significantly impede their daily activities and overall well-being. Furthermore, the substantial economic burden imposed by pathological diseases on healthcare systems, governments, and individuals encompasses direct costs from medical treatments and hospitalizations, coupled with indirect costs related to productivity loss and disability, leading to significant financial strain. The impact of these diseases extends beyond the physical realm, with affected individuals often facing social stigma, isolation, and mental health challenges such as anxiety and depression, intensifying the overall burden of the disease. Moreover, the prevalence of pathological diseases strains healthcare systems, resulting in heightened demand for resources, specialized care, and medical interventions, leading to overcrowded healthcare facilities, longer wait times, and challenges in providing adequate and timely care for all patients. To address these complex challenges, a concerted effort is being made towards preventive measures, early detection, effective treatments, and holistic healthcare approaches. Public health initiatives, education, and awareness campaigns are pivotal in promoting healthy behaviors, reducing risk factors, and enhancing overall disease management, while the development of innovative and personalized treatments, along with the integration of technology in healthcare delivery, remains essential for improving the well-being of individuals and communities affected by pathological diseases.

### 4.2 Current treatment for better cure

Pathological diseases encompass a wide range of conditions affecting various organs and systems in the body, and treatment approaches often depend on the specific disease and its underlying causes. For cancer, treatment can involve surgery, chemotherapy, radiation therapy, targeted therapy, immunotherapy, and hormone therapy, with advancements in personalized medicine leading to more targeted and effective treatments based on specific genetic mutations and tumor characteristics. Cardiovascular diseases may necessitate lifestyle changes, medications, interventional procedures, or surgeries, while neurological disorders may be managed through medications, therapy, and sometimes surgical interventions, with targeted therapies emerging for neurodegenerative diseases like Alzheimer's and Parkinson's. Autoimmune diseases often require immunosuppressant medications, biological therapies, and lifestyle modifications. Treatments for infectious diseases encompass a wide range of medications and therapies, including antibiotics, antiviral drugs, antifungal medications, vaccines, monoclonal antibodies, and antiviral therapies for specific infections such as HIV, hepatitis C, and COVID-19. In the case of genetic disorders, while many do not have a cure, treatments focus on managing symptoms and improving quality of life through medications, gene therapies, enzyme replacement therapies, and in some cases, stem cell transplantation. It's crucial to note that medical advancements are continuously evolving, and new treatments may have emerged since my last update, so consulting a healthcare professional or referring to recent medical literature for the most accurate and up-to-date information is recommended.

## 5 Limitations

Despite the wide array of available treatments for pathological diseases, there are certain limitations to these approaches. While surgery, chemotherapy, and radiation therapy are effective for many cancers, they can be physically and emotionally taxing for patients and may not always guarantee complete remission or prevent relapse. Similarly, targeted therapies in personalized medicine are often contingent upon specific genetic mutations, limiting their efficacy for patients with different tumor characteristics. Lifestyle changes and medications for cardiovascular diseases can help manage the condition, but they may not fully reverse the underlying pathology, and some patients may still experience disease progression. In the case of neurological disorders, although therapies and medications can alleviate symptoms, they may not halt disease progression, leaving patients with a continued decline in neurological function. Furthermore, treatments for autoimmune diseases, while helpful in controlling symptoms, may lead to increased susceptibility to infections and other complications due to the suppression of the immune system. For infectious diseases, the emergence of drug-resistant pathogens poses a significant challenge, limiting the efficacy of antibiotics and antiviral medications. Additionally, the development and accessibility of vaccines for certain infectious diseases may be constrained by logistical, socioeconomic, and geopolitical factors. While treatments for genetic disorders can improve the quality of life for patients, they often target symptoms rather than address the root cause, and not all patients may have access to or be suitable candidates for advanced therapies such as gene therapies or stem cell transplantation. Recognizing these limitations underscores the ongoing need for continued research and development of more comprehensive and accessible treatment modalities for pathological diseases.

### 5.1 Need of this study

This review article is needed to reconnoitre the therapeutic potential of honey infused with herbs in treating pathological diseases. It aims to assess the scientific evidence supporting this natural remedy, highlighting the individual benefits of honey and selected herbs, their synergistic effects, and potential mechanisms of action. The article will also address safety considerations and the need for further research and clinical trials to unlock the full potential of this approach in medical treatments.

### 5.2 The concept of honey infused with herbs as a potential breakthrough in holistic healthcare

Infused honey serves as a versatile culinary ingredient, contributing a distinctive and delightful flavor spectrum to an array of dishes and beverages, whether it imparts a fiery zest to a marinade or introduces a subtle floral essence to a comforting cup of tea. Furthermore, the infusion process has the potential to enhance the health benefits of honey by incorporating herbs, spices, or fruits, thereby augmenting its medicinal properties. For instance, infusing honey with turmeric can elevate its anti-inflammatory characteristics, while the addition of lemon may potentially reinforce its immune-boosting effects. Additionally, using infused honey as a natural sweetener provides a seamless substitution for sugar in a variety of recipes, including dressings, sauces, and baked goods, offering a healthier alternative without compromising on taste.

The pro-health properties of infused honey stem from the presence of biologically active compounds, encompassing secondary metabolites of plants such as phenolic acids, flavonoids, coumarins, tannins, lignans, and terpenoids [6]. In honey, these compounds derived from nectar, pollen, propolis, and honeydew are utilized to determine the botanical origin of the honey [7]. Notably, herb-infused honey exhibits robust health-promoting attributes, particularly in terms of antimicrobial and antioxidant activity, distinguishing it from regular nectar honey [[8], [9], [10]].

Efficient fortification of honey with herbs through prolonged maceration of flowers or leaves demonstrates a higher efficacy compared to the process involved in herb honey production. While macerating flowers in honey enhances its antioxidant content, the significantly elevated concentration of herbal ingredients yields even more pronounced results. The weak correlation between antioxidant activity and the herbal ingredient content suggests that the bioactive components do not solely dictate the antioxidative activity of honey. In conclusion, fortifying honey with plants yields a novel product with promising therapeutic potential for the treatment and prevention of conditions such as lymphedema and chronic venous disease [11].

## 6 The resurgence of interest in natural remedies and alternative therapies in modern times

Traditional Chinese, Ayurveda, Kampo, Korean, and Unani medicine systems, which have evolved into meticulously regulated systems, demonstrate the importance of natural products and traditional medicines. The use of nutraceuticals, phytonutrients, and herbal remedies is expanding quickly throughout the world as more people turn to these items to treat a range of health issues under various national healthcare systems [12]. In both emerging and established nations, there has been a noticeable upsurge in the last ten years in the acceptance and public interest in natural medicines. These herbal cures are now widely available not just in drugstores but also in food shops and supermarkets. An estimated four billion people, or 80 % of the world's population who live in poorer nations, depend mostly on herbal medical goods for their healthcare needs. Furthermore, these groups view traditional herbal medicine practises as an essential component of their culture [[13], [14], [15]]. Complementary and alternative medicines (CAMs) are becoming commonplace in the UK, the rest of Europe, North America, and Australia as a result of the extensive adoption of herbal remedies in many developed nations (Committee on the Use of Complementary and Alternative Medicine by the American Public, Board on Health Promotion, and Disease Prevention, Institute of Medicine, 2005 [[16], [17], [18]], several other European nations also have a well-established and widespread use of herbal medicines [16]. Among many other reasons, one of the main reasons people seek out herbal medicines in these industrialized nations is the conviction that using herbal therapy promotes a healthier lifestyle. As a result, people tend to view herbal medications as a reasonable and well-rounded approach to healing, which has resulted in them spending large amounts of money—billions of dollars—on over-the-counter and home herbal remedies. The notable increase in sales of herbal medicines, which account for a sizeable share of the worldwide pharmaceutical market, can be attributed to this trend. With so many new products hitting the market and the world using herbal medicines for medical purposes, worries about their safety and public health are becoming more widely acknowledged. While some herbal remedies are widely used and have intriguing potential, many are still unproven, and their use is not closely controlled. As a result, little is known about their possible side effects, which makes it difficult to determine which treatments are the safest and most effective and to encourage their sensible application [19]. Moreover, it is often recognized that insufficient labelling, improper quality controls, and a lack of pertinent patient information further jeopardize the safety of the majority of herbal medicines [20]. As a result, it is now crucial to give the general public including medical professional enough information to improve their comprehension of the risks connected to these items and to guarantee that all medications are secure and of the right calibre.

### 6.1 Nutritional composition

**Fig. 1:**
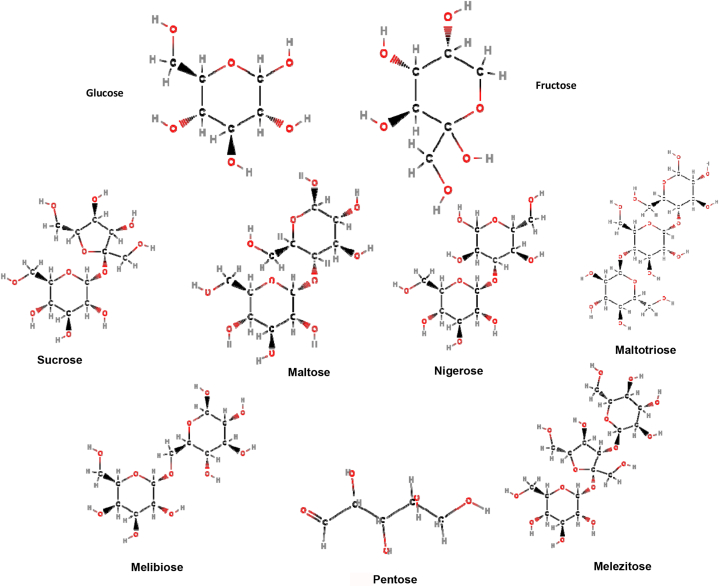
Chemcial Structures of Honey Sugars Nutritional content of honey (per 100g) includes Carbohydrates 300 kcal, Proteins 0.5 g,Sodium 1.6–17 mg, Calcium 3–31 mg, Potassium 40–3500 mg, Magnesium 0.7–13 mg, Phosphorus 2–15 mg, Zinc 0.05–2 mg, Copper 0.02–0.6 mg, Iron 0.03–4 mg, Manganese 0.02–2 mg, Chromium 0.01–0.3 mg, Selenium 0.002–0.01 mg, Phyllochinon (vitamin K) 0.025 mg, Thiamine (vitamin B_1_) 0.00–0.01 mg, Riboflavin (vitamin B_2_) 0.01–0.02 mg, Pyridoxin (vitamin B_6_) 0.01–0.32 mg, Niacin 0.10–0.20 mg, Pantothenic acid 0.02–0.11 mg, Ascorbic acid (vitamin C) 2.2–2.5 mg [22].

Honey is a natural sweetener and source of energy about 64 calories per tablespoon. It is made up of carbohydrates (80–90 %) and water (17.1 %). Water is an essential component of honey. The main carbohydrate components of honey are glucose and fructose which represent 80–95 % of total sugars. The remaining sugar comprises disaccharides such as sucrose, maltose, pentose, maltotriose, melezitose, nigerose and melibiose (Fig. 1). Small amounts of oligosaccharides are also present. It contains 4–5% fructo-oligosaccharides, which have no pathogenicity and a positive effect on health. Honey contains 0.57 % of organic acids accountable for the taste, and acidity. It contains0.1 %–1.0 % of minerals like K, Ca, Mg, Na, S, and P, and trace elements like Fe, Cu, Zn, and Mn. Honey contains nitrogenous compounds, vitaminB_1_, B_2_, complex, vitamin B_6,_and vitamin C. It has proteins only in minute quantity i.e., 0.1–0.5 %. Honey is composed of a large number of enzymes viz. invertase, catalase, amylase, oxidase, etc. [3,21].

### 6.2 Therapeutic health effects of honey

Honey possesses antioxidant properties due to the presence of flavonoids and phenolic acids; these eradicate free radicals from the body. Honey exhibits lots of biological activities like anti-inflammatory, anti-thrombotic, immune boosting, antibacterial, antiviral, anti-allergic and vasodilatory activities. It is beneficial for diarrhoea, gastric ulcer, arthritis, diabetes mellitus, ophthalmology, cough, fungal infections, wound healing, gastrointestinal disorders, immunity boosters, and cancer diseases [2,23].

### 6.3 Honey and herbs

Honey is a proven wound healer, used by human civilizations since time immemorial, and is still in use in many places in the world. When infused with medicinal herbs, its potential increases many fold to cure many ailments.

## *7* Allium sativum L

*Allium sativum* L, commonly known as garlic or Lahsun, belongs to the Kingdom Plantae and the Division Magnoliophyta. It is classified under the Order Asparagales, the Family Amaryllidaceae, and the Genus *Allium*, with the specific species name being *sativum*. Garlic, or Lahsun, is a well-known plant with a long history of culinary and medicinal uses, and its taxonomic classification places it within the broader context of plant taxonomy and classification.

**Distribution:***Allium sativum* is a perennial plant having its origin in Asia. India, China, Turkey, Spain, Egypt, and South Korea are the largest producers of garlic. It is used for medicinal, culinary, and antimicrobial purposes. In India, it is cultivated throughout the country [24,25].

**Fig. 2:**
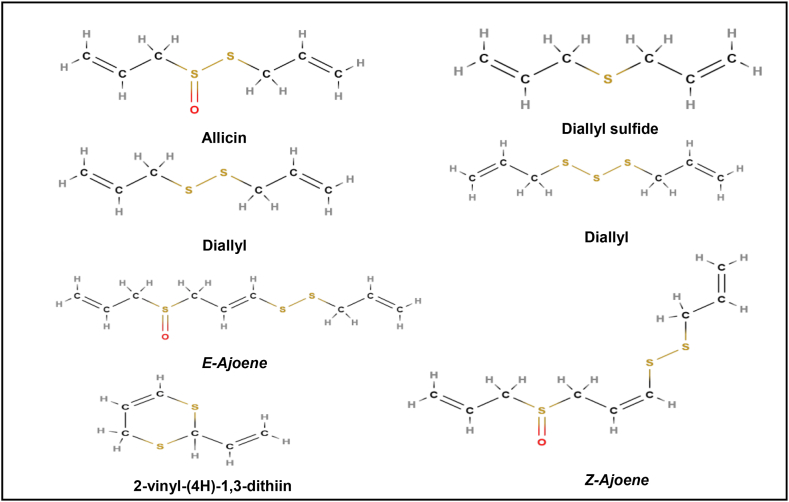
Active compounds of Allium sativum.

**Active constituents:** The bulbs of Allium sativum contain compounds containing sulfur viz. thiosulfates (allicin), vinyldithiins (2-vinyl-(4H) −1,3-dithiin,3-vinyl-(4H)-1,2-dithiin), ajoenes (E-ajoene, Z-ajoene), sulfides (diallyl disulfide, diallyl trisulfide, and others) (Fig. 2). Allicin (allyl thiosulfinate) is a sulfenic acid thioester which is having strong antioxidant activity [26].

Pharmacological Effects: *Allium sativum*has strong activity against protozoal, viral, inflammation, cancer, hypertension, diabetes, and bacteria etc. It also possesses fungicidal, cardiovascular, hepatoprotective and antibacterial properties that make it a proven medicinal plant (Table 3) [26,27].

**Table 3:** Wound healing property of aqueous extract of *Allium sativum* fortified with honey.

Model	Method	Intervention (Activity)	Outcome	Reference
Sprague dawley rats	18 Sprague dawley wounded rats were taken Group 1: Alone Honey was applied Group 2: Honey + garlic extract Group 3: Solcoseryle-jelly.	On day 3 and day 7 after wound induction, swabs were taken from the wound surface and cultured on Brain Heat Infusion (BHI) agar at 37 °C overnight for any bacterial growth. It was demonstrated that there was no bacterial growth on days 3 and 7. The results indicated antimicrobial compounds in honey alone, honey in combination with garlic extracts as well as in solcoseryle-jelly. The wound healing is faster in Group 2 followed by Group 1 and Group 3.	The extract of *Allium sativum* when combined with honey proved beneficial in healing wounds.	[28]

## *8* *Alpinia galanga* (L.)Willd

*Alpinia galanga* (L.)Willd., commonly known as Galanga, is classified within the Kingdom Plantae and the Division Magnoliophyta. It is categorized under the Order Zingiberales, the Family Zingiberaceae, and the Genus Alpinia, with the specific species name being galanga. Galanga is a plant with notable significance in various cultures, commonly utilized for its culinary and medicinal properties. Its taxonomic classification highlights its position within the broader framework of plant classification, emphasizing its association with the Zingiberaceae family and the Zingiberales order.

**Distribution:***Alpiniag alanga* grows in forests and open spaces in many countries of Asian continent such as China, Sri Lanka, India, Arabia, and Indonesia. It has also been reported inthe Indian Himalayan regions and Western Ghats [29].

**Fig. 3:**
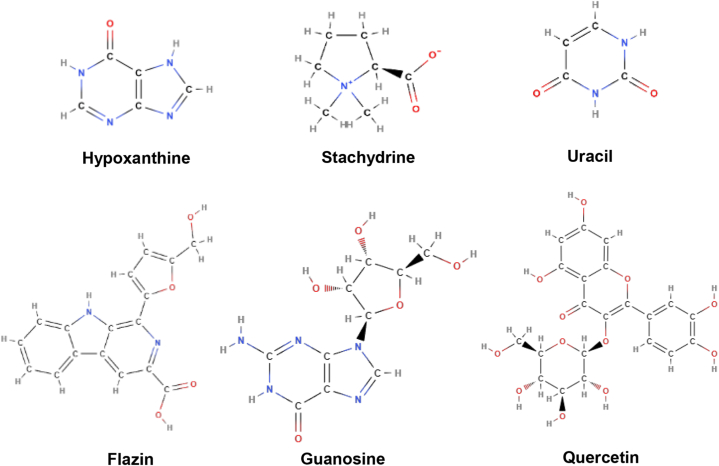
Active compounds of *Alpinia galanga*.

**Active constituents:** The key active compounds found in *A. galanga* are 1,8-cineol, α-bergamotene, α-fenchyl acetate, β-bisabolene, β-farnesene, β-pinene, 1′-acetoxychavicol acetate, galango flavonoid, 1′S-1′-acetoxychavicol acetate (ACE), phenyl propanoids and phydroxybenzaldehyde (1′S-1′-acetoxychavicol acetate and 1′S-1′-acetoxyeuginol acetate), acetoxycineoles (trans and cis)-2-and 3-acetoxy- 1, 1, 8-cineoles, 1′-acetoxychavicol acetate (galangal acetate), β-sitosterol diglucoside (AG-7) and β-sitsteryl arabinoside (AG-8), hydroxy-1,8-cineole glucopyranosides, (1R, 2R, 4S)-and (1S, 2S, 4R)-trans-2-hydroxy-1,8-cineole β-d-glucopyranoside, and (1R, 3S, 4S)-trans-3-hydroxy-1, 8-cineole β-d-glucopyranoside (Fig. 3) [30].

**Pharmacological Effects:** Alpinia galangal possesses antifungal, antimicrobial, anti-inflammatory, anti-hepatotoxic, anti-HIV, anti-SARS-CoV-2, immunomodulatory, anti-diabetic, anti-ulcer, anti-tumour, anti-allergic and antioxidant activities (Table 4) [29].

**Table 4:** *Alpinia galanga* in combination with honey and different type of herbs used for the treatment of mild asthma in human beings.

Model	Method	Intervention (Activity)	Outcome	Reference
Asthmatic patients	80 patients with mild asthma were selected, the control was given fluticanose/salbutamol standard drugs for treating asthma and the experimental were given honey syrup which was made by mixing honey and extract of *Alpinia galanga,* cinnamon, saffron, cardamom, and ginger. Total scores of ACQ (Asthma Control Questionnaire) were evaluated.	After 8 weeks of study, experimental groups given compound honey syrup decreased asthma-associated symptoms like shortness of breath, activity limitation, and wheezing.	Results demonstrated that compound honey syrup is an effective and safe formulation for treating paediatric asthma and associated symptoms.	[31]

## *9* *Capparis spinosa* L

*Capparis spinosa* L., commonly known as the Caper bush or Himsara, is classified within the Kingdom Plantae and the Division Magnoliophyta. It falls under the Order Capparales, the Family Capparaceae, and the Genus Capparis, with the specific species name being spinosa. The Caper bush, also referred to as Himsara, is a notable plant with diverse applications, ranging from culinary to medicinal purposes. Its taxonomic classification underscores its position within the Capparaceae family and the Capparales order, providing valuable insight into its botanical categorization within the broader context of plant taxonomy.

**Distribution**: It is a spiny shrub endemic to the Mediterranean basin, disseminated in Northern and Eastern Africa, Southern Europe, South–Western and Central Asia, Papua New Guinea, Indonesia, Australia, and Oceania [32].

**Fig. 4:**
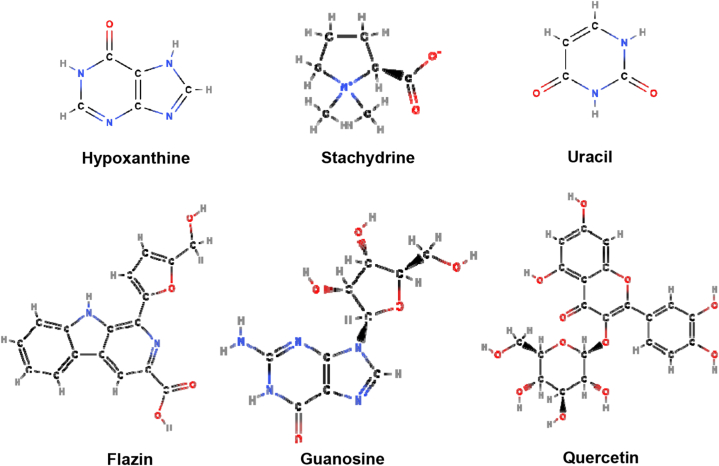
Active compounds of Capparis spinosa.

**Active constituents:** The chemical compounds available in *Capparis spinosa* include alkaloids, terpenoids, flavonoids, glucosinolates, phenolic acids, etc. The main components of its aerial parts are cappariloside A, stachydrin, hypoxanthine, uracil, capparine A, capparine B, flazin, guanosine, 1*H*-indole-3-carboxaldehyde, 4-hydroxy-1*H*-indole-3-carboxaldehyde, kaempferol, thevetiaflavone, tetrahydroquinoline, rutin, kaempferol3-glucoside, kaempferol-3-rutinoside, kaempferol-3-rhamnorutinoside, isorhamnetin 3-*O*-rutinoside, quercetin 3-*O*-glucoside, ginkgetin, isoginkgetin, sakuranetin and glucocapparin in aerial parts. The main components of root are capparispine, cadabicine 26-*O*-β-D-glucoside, capparispine 26-*O*-β-D-glucoside, and stachydrine, seeds contain glucocapparin(Fig. 4) [33].

**Pharmacological Effects:** The plant possesses antibacterial, anti-carcinogenic, anti-arthritic, antioxidant, anti-quorum sensing and antibiofilm potential, antifungal, analgesic, Immunomodulatory, antidiabetic, nematocidal, antispasmodic, hepatoprotective, anti-inflammatory, neuroprotective and bone regeneration property (Table 5) [32,33].

**Table 5:** Therapeutic potential of the herbal formulation of *C. spinosa* combined with honey used for improving the toxic effects of trichloroacetic acid.

Model	Method	Intervention (Activity)	Outcome	Reference
Swiss albino male mice intoxicated with trichloroacetic acid (TCA).	120 male albino mice were selected. Group I: It was treated with distilled water (4 ml/kg for 3 and 6 weeks) and kept as control. Group II: It was treated orally with honey at a dose of 4 ml/kg for 3 consecutive weeks. Group III: It wasgivencombination of *Capparis spinosa* leaf powder fortified with honey (40 mg/kg body weight) for 3 weeks. Group IV: It was treated with extract of *Capparis spinosa* leaf powder alone orally for 3 weeks. Group V:It was given TCA alongwith drinking water (500 mg/kg), and then left to recover for 3 weeks. Group VI: It was given TCA for 6 weeks and then given a formulation of honey &*Capparis spinosa* for 3 weeks.	The group treated alone with TCA for 6 weeks caused 6 % mortality in the mice. The death rate rose to 14 % in the mice left for recovery.The least mortality was reported in the mice given TCA followed bythecombination of *Capparis spinosa* and honey. This group also exhibiteda significant increase in the final body weight.	The results indicate that a mixture of leaf powder of *Capparis spinosa* and honey at a prescribed dose (40 mg/kg body weight) for 3 weeks improved the toxicity caused by trichloroacetic acid and improved both histopathological lesions, biochemical attributes and decreased percentage of mortality as well.	[34]

## *10* *Curcuma xanthorrhiza*Roxb

*Curcuma xanthorrhiza* Roxb., commonly referred to as Javanese turmeric, belongs to the Kingdom Plantae and the Division Magnoliophyta. It is classified under the Order Zingiberales, the Family Zingiberaceae, and the Genus *Curcuma*, with the specific species name being *xanthorrhiza*. Javanese turmeric is a notable plant renowned for its various applications, both in culinary and medicinal contexts. Its taxonomic classification highlights its association with the Zingiberaceae family and the Zingiberales order, offering valuable insights into its botanical placement within the broader domain of plant taxonomy.

**Distribution:**It is native to Indonesia, and widely distributed in Java, Kalimantan, Sulawesi, Sumatra, and Maluku. *Curcuma xanthorrhiza is* grown in Southeast Asia including Thailand, Malaysia, Vietnam,and the Philippines. It is also distributed in Japan, China, India, and Korea [35].

**Fig. 5:**
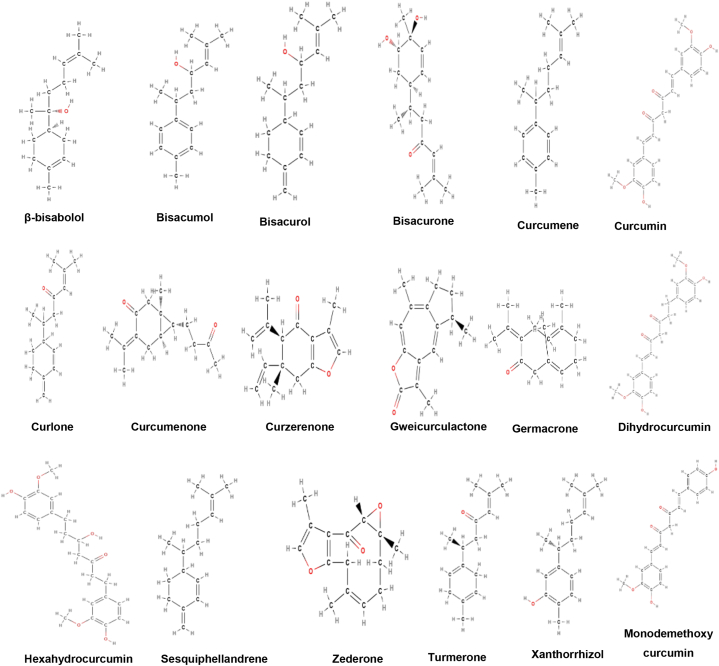
Active compounds of *Curcuma xanthorriza*.

**Active constituents:** The active compounds of *Curcuma Xanthorriza* are curcumin, bisacurone, curlone, α-curcumene, bisacumol, bisacurol,arturmerone, xanthorrhizol,β sesquiphellandrene, curzerenone, germacrone,β-curcumene,α-turmerone, β-turmerone,(−)-curcuhydroquinone 2,5-di-*O*-β-d-glucopyranoside, β-bisabolol, zedoardiol,3,4 dihydroxybisabola-1,10-diene, 13-hydroxy-xanthorrhizol,12,13-epoxy-xanthorrhizol,zedoarol, zederone, curcumenone,zedoaral

dehyde, gweicurculactone, dihydrocurcumin, hexahydrocurcumin, monodemethoxycurcumin,bis-demethoxy curcumin, etc. (Fig. 5) [35].

**Pharmacological Effects:** Curcuma xanthorriza exhibits antioxidant, immunomodulatory antibacterial, antifungal, anticancer, antitumor, antidiabetic, hepatoprotective, insect repellent, and anti-inflammatory properties (Table 6) [35,36].

**Table 6:** Therapeutic potential of herbal honey formulation added with *Curcuma xanthorriza* and black cumin against cancer cells.

Model	Method	Intervention (Activity)	Outcome	Reference
Sprague Dawley (SD) Rats and human mammary cancer cells	The anticancerous activity of *Curcuma xanthorriza*, and black cumin mixed with honey were tested on dimethylbenzene(*a*)anthracene induced rats.	The herb infused honey formulation inhibited the formation of tumor nodules, and enhanced the glutathione-S-transferase activity along with an increase in CD4, CD8, and CD4 and CD25 cells.	The formulation showed anti cancerous and immunomodulatory activities.	[37]

## *11* *Euphorbia hirta* L

*Euphorbia hirta* L., commonly known as the Asthma plant or Dhudika, is classified within the Kingdom Plantae and the Division Tracheophyta. It is categorized under the Order Malpighiales, the Family Euphorbiaceae, and the Genus *Euphorbia*, with the specific species name being *hirta*. The Asthma plant, or Dhudika, is a significant botanical specimen known for its medicinal properties and traditional uses in treating respiratory ailments. Its taxonomic classification underscores its affiliation with the Euphorbiaceae family and the Malpighiales order, providing valuable insights into its botanical categorization within the broader context of plant taxonomy.

**Distribution:***Euphorbiahirta* is native to central tropical America, however, inhabits hot areas all over Australia and India. It is generally found in barren areas near the roadside [38].

**Fig. 6:**
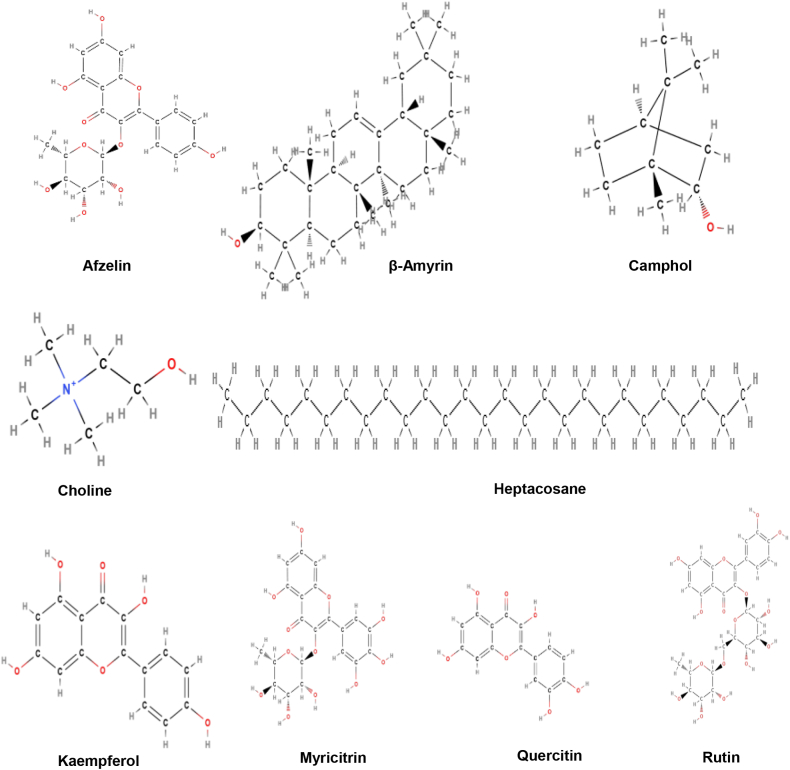
Active compounds of *Euphorbia hirta*.

**Active constituents:** Various active constituents of *E. hirta*include Afzelin, quercitrin, myricitrin, rutin, quercitin, euphorbin-A, euphorbin-B, euphorbin-C, euphorbin-D,2,4,6-tri-*O*-galloyl-β-d-glucose,1,3,4,6-tetra-*O*-galloyl-β-d-glucose, gallic acid, kaempferol, protocatechuic acid, β-amyrin, 24-methylenecycloartenol, nonacosane, heptacosane, β-sitosterol, tinyatoxin, shikmic acid, choline, camphol, and quercitol derivatives containing rhamnose and chtolphenolic acid (Fig. 6) [39].

**Pharmacological Effects:** The plant possesses anti-malarial, galactogenic, anti-asthmatic, anti-inflammatory, antidiarrheal, antioxidant, antifertility, antiamoebic, antifungal, and anti-diuretic activities (Table 7).

**Table 7:** Therapeutic potential of the herbal preparation of *Euphorbia hirta* and honey against stomach ulcer.

Model	Method	Intervention (Activity)	Outcome	Reference
Albino wistar rats	45 healthy albino wistar male rats were selected for study that was divided in desired numbers of groups. Group 1 was given only water and kept as negative control. Group 2 was treated with misoprostol and kept as the positive control. Groups 3, 4 & 5 were given extract of *Euphorbia hirta* in the conc. of 0.2,0.4, and 0.8 gm/kg body weight respectively. Groups 6, 7 & 8 was treated with a formulation of honey mixed with *Euphorbia hirta*extract (200, 400 and 800 mg/kg). Group 9 was given 1 ml of honey alone.	Least ulceration activity was demonstrated by Group 6 to 8 which were given honey infused herbs followed by other groups.	The results indicate that *Euphorbiahirta* combined with honey has antiulcer activities against as tested in rats.	[40]

## *12* *Nigella sativa*L

*Nigella sativa* L., commonly known as Black cumin or Kalonji, belongs to the Kingdom Plantae and the Division Magnoliophyta. It is classified under the Order Ranunculales, the Family Ranunculaceae, and the Genus Nigella, with the specific species name being sativa. Black cumin, or Kalonji, is a notable plant widely recognized for its culinary and medicinal significance, particularly in traditional medicine systems. Its taxonomic classification highlights its association with the Ranunculaceae family and the Ranunculales order, emphasizing its position within the broader context of plant taxonomy.

**Distribution:***Nigella sativa* is an angiospermic plantendemic to Southwest Asia. Earlier, it was localized to the Mediterranean region only but is now available in Himachal Pradesh, Bihar, Assam, Jammu-Kashmir, and Punjab states as well. It is also cultivated in North-EastIndia and Bengal [41].

**Fig. 7:**
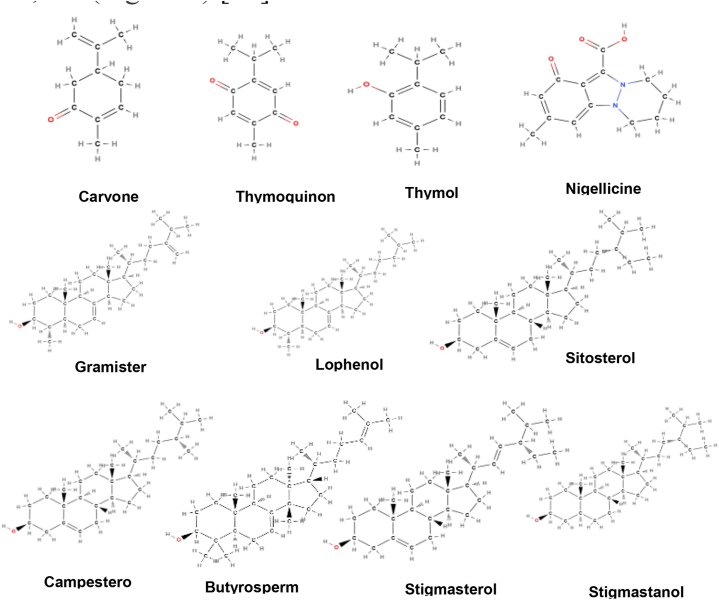
Active compounds of *Nigella sativa*.

**Active Constituents:** Seeds and seed oil of *Nigella sativa* contain certain phytochemicals like carvone, nigellone, thymoquinone, thymol, nigellicine, nigellicimine, nigellicimine N-oxide, cholesterol, campesterol, gramisterol, lophenol, sitosterol, stigmastanol, stigmasterol, beta-amyrin, butyrospermol, oleic acid, esters of unsaturated fatty acids, tannins, glycoside, resin, glycosidal saponin, etc.(Fig. 7) [42].

**Pharmacological Effects:** The plant Nigella sativa exhibits antidiabetic, immunomodulator, bronchodilator, spasmolytic, hepatoprotective, anti-inflammatory, antimicrobial, gastro-protective, antioxidant, antifertility, anticancer, cytotoxic, wound healing, analgesic and antihelmintic properties (Table 8) [43,44].

7. *Phyllanthus emblica* L.

**Table 8:** Therapeutic potential of *Nigella sativa* in combination with honey, beneficial for wound healing purposes in rats.

Model	Method	Intervention (Activity)	Outcome	Reference
Wistar rats	50 rats were divided into desired number of groups: I. Control: lanolin was administered II. Honey group: administration of honey III. *Nigella sativa* group: cold-pressed *Nigella sativa* seed oil IV.Mixgroup: mix of honey and *Nigella sativa* seed oil (1:1 ratio) V. Phenytoin group: Phenytoin cream was administered.	Wound healing was maximum in group IV as evidenced by decrease in wound area after excision.	The use of topical formulation prepared from honey and *Nigella sativa* seed oilimproved and accelerated the wound healing.	[45]

*Phyllanthus emblica* L., commonly known as Amla or Indian gooseberry, is classified within the Kingdom Plantae and the Division Magnoliophyta. It falls under the Order Malpighiales, the Family Phyllanthaceae, and the Genus *Phyllanthus*, with the specific species name being *emblica.* Amla, or Indian gooseberry, is a renowned plant celebrated for its diverse applications in both culinary and traditional medicinal practices. Its taxonomic classification underscores its affiliation with the Phyllanthaceae family and the Malpighiales order, providing valuable insights into its botanical categorization within the broader context of plant taxonomy.

**Distribution:***Phyllanthus emblica*is a deciduous tree, that originated in India and is grown in many countries like China, Malaysia, Bangladesh, Myanmar, Sri Lanka, Pakistan, and Uzbekistan [46].

several other tropical and sub-tropical countries (Fig. 1)

such as Bangladesh, China (southern part), Malaysia, Mas-carene Islands, Myanmar, Pakistan, Sri Lanka, and Uzbeki-stan.

**Fig. 8:**
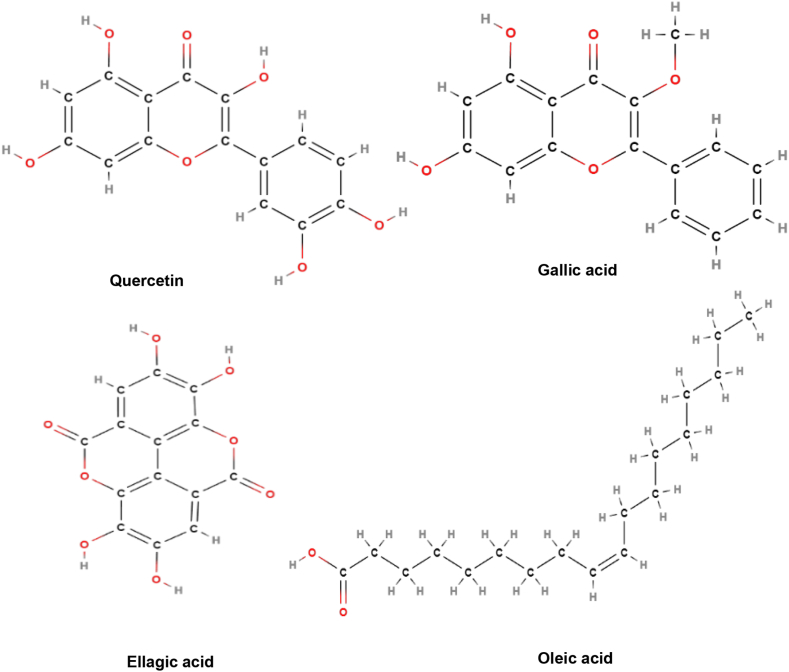
Active compounds of *Phyllanthus emblica*.

**Active constituents:** Fruits of *Phyllanthus emblica* contain ellagic acid, fibers, nicotionic acid, gallic acid, carbohydrates, proteins, fats, minerals, phyllem-belin and phyllembelic acid. Seeds contain linolenic acid, oleic acid, linoleic acid, palmitic acid, stearic acid, and myristic acid. The leaves contain chebulic acid, gallic, ellagic, chebulagic, chebulinic, and amlic acid, alkaloids phyllantidine, and phyllantine. Roots of the plant contains ellagic acid and lupeol. The bark contains leucodelphinidin [47]. Other active constituents of Amla include 1-*O*-galloyl-beta-d-glucose, 3,6-di-*O*-galloyl-d-glucose, corilagin, 1,6-di-*O*-galloyl beta D glucose, quercetin, chebulagic acid, chebulinic acid, 3-ethylgallic acid (3-ethoxy-4, 5-dihydroxy benzoic acid) and isostrictiniin (Fig. 8) [48].

**Pharmacological Effects:***Phyllanthus emblica* is used in the folk system of medicine for curing different disorders likediarrhea, diabetes, and dysentery. It possesses strong antioxidant, antidiabetic, antibacterial, antiulcerogenic, chemopreventive, hypolipidemic, gastroprotective, andhepatoprotectiveactivities(Table 9) [47].

8. Sesamum indicum L.

**Table 9:** Therapeutic potential of *Phyllanthus emblica* in combination with honey used for the treatment of gastroesophageal reflux disease.

Model	Method	Intervention (Activity)	Outcome	Reference
Albino wistar rats	1. In vitro anti-spasmodic activity: Group I: treated with Acetylcholine and normal saline. Group II: Rats treated with Acetylcholine given Honey Amla formulation (25 μg/mL). Group III: Rats treated with Acetylcholine given Honey Amla formulation (50 μg/mL dose). Group IV: Rats treated with Acetylcholine given Honey Amla formulation (100 μg/mL dose). 2. Charcoal Meal Motility (CMM) Test: Group I: N saline. Group II: Treated with Rebamipide and Pantoprazole. Group III: Treated with Amla and Honey. Group IV: Treated with Rebamipide, Pantoprazole with Amla and Honey.	1. In vitro antispasmc activity test revealed that contractility pattern was lowered in Amla and Honey's formulation in dose dependent manner against acetylcholine. 2. In CMM Test, Group given amla honey formulation along with Rebamipide, Pantoprazole showed lower gastrointestinal motility	The herbal combination of *Phyllanthus emblica* and Honey in comparison with other groups declines the Gastroesophageal reflux issue, and anti-spasmodic activity was also exhibited in ileum of treated rat. Overall, reflux was controlled in rats.	[48]

*Sesamum indicum* L., commonly known as Sesame, is classified within the Kingdom Plantae and the Division Tracheophyta. It is categorized under the Order Lamiales, the Family Pedaliaceae, and the Genus *Sesamum*, with the specific species name being *indicum*. Sesame is a well-known plant with significant agricultural, culinary, and medicinal importance. Its taxonomic classification highlights its association with the Pedaliaceae family and the Lamiales order, providing valuable insights into its botanical placement within the broader context of plant taxonomy.

**Distribution:** It is an oilseed crop used all over the world for nutritional as well as medicinal purposes. It is cultivated throughout the plains of India [29,49].

**Fig. 9:**
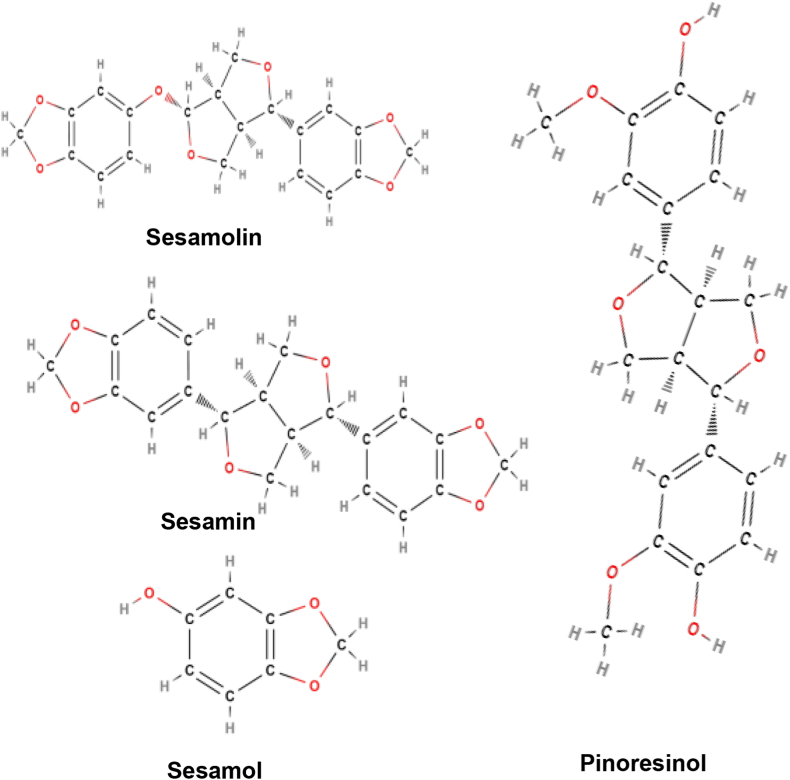
Active compounds of *Sesamum indicum*.

Active constituents: Phytochemical compounds isolated from different parts of sesame are lignans, phenols, polyphenols, phytosterols, triterpenoids, anthraquinones, naphthoquinones, aldehydes and other organic compounds. Lignans are the main constituents in seeds of sesame, responsible for strong antioxidant activity. Lignans include sesamin, sesamolin, sesamol, (+)-episesaminol 6- catecho, (+)-episesaminone, pinoresinol, (−)-pinoresinol 4-*O*-glucoside, (+)-pinoresinol di-*O*-β-d-glucopyranoside, sesaminol, (+)-sesaminol 2-*O*-β-D-glucoside, (+)-sesaminoldiglucoside, sesamolinol, (+)-sesamolinol4′-*O*-β-D-glucoside, samin, matairesinol, sesangolin and disaminyl ether (Fig. 9) [50].

**Pharmacological Effects:***Sesamum indicum* exhibits various therapeutic properties that prove its pharmaceutical significance such as antimicrobial, anti-inflammatory, antidiabetic, antioxidant, anti-cancerous, antihyperlipidemic, anthelmintic, antileishmanial, gastroprotective, and vasorelaxant activities (Table 10) [51].

9. Spinacia oleracea L.

**Table 10:** Therapeutic potential of *S. indicum* oil combined with honey and camphor oil used as an herbal ointment for the second-degree burn in rats.

Model	Method	Intervention (Activity)	Outcome	Reference
Wistar-albino male rats	40 rats were selected: Group 1: Treated with formulation made of sesame oil, camphor, and honey. Group 2: Given Vaseline treatment.	It was demonstrated that epithelization and neovascularization wereregulated by formulation	Significant healing was observed by applying formulation fortified with honey.	[52]

*Spinacia oleracea* L., commonly known as Palak or Spinach, is classified within the Kingdom Plantae and the Division Magnoliophyta. It is categorized under the Order Caryophyllales, the Family Amaranthaceae, and the Genus *Spinacia*, with the specific species name being *oleracea.* Spinach, a popular leafy green vegetable, is celebrated for its nutritional value and versatile culinary applications. Its taxonomic classification emphasizes its affiliation with the Amaranthaceae family and the Caryophyllales order, offering valuable insights into its botanical categorization within the broader context of plant taxonomy.

**Distribution:** Spinacia *oleracea* has been reported to be domesticated in Iran and former Persia, about 2000 years ago. However, it is also now grown in China, Nepal, India, Pakistan, and Afghanistan [53].

**Fig. 10:**
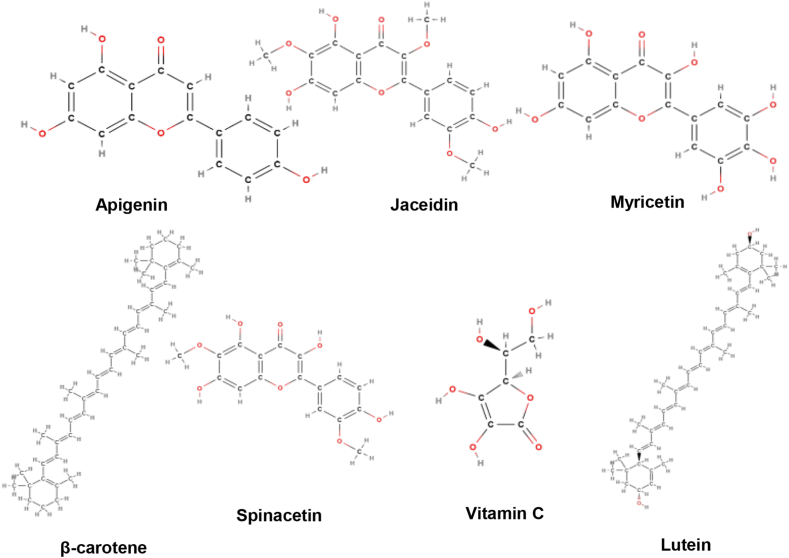
Active compounds of *Spinacia oleracea*.

**Active constituents:***Spinacia oleracea* contains various types of secondary metabolites like phenolic compounds, carotenoids, and flavonoids. The phenols isolated from the plant are ferulic acid, *para*-coumaric acid, and *ortho*-coumaric acid. Different carotenoids are β-carotene, lutein, violaxanthin, and 9'-(Z)-neoxanthin. Flavonoids like spinacetin; 5,3′,4′**-**trihydroxy-3-methoxy 6,7-methylenedioxyflavone-4′-glucuronide; jaceidin; kampeferol; 5,4′-dihydroxy-3.3′-dimethoxy-6,7-methylene dioxyflavone-4′-glu-curonide; querecetin, myricetin; 5,4′-dihydroxi-3,3′-dimithoxi-6,7-methylene-dioxi-flavones; apigenin and luteolin are present. It contains high concentrations of vitamins A, C, E, K, oxalic acid, and folic acid. Presence of various types of minerals like magnesium, manganese, iron, zinc, calcium, phosphorus, copper, and potash (Fig. 10) [54].

**Pharmacological Effects:** The plant possesses antioxidant, antiproliferative nerve depressant, antihistaminic, anticancer, anti-inflammatory and hepatoprotective properties (Table 11) [55].

10. *Tinosporacordifolia*(Willd.)

**Table 11:** Therapeutic potential of *Spinacia oleracea* incombination with *Solanum lycopersicum* and honey for the treatment of anaemic conditions in pregnant women.

Model	Method	Intervention (Activity)	Outcome	Reference
Pregnant women	Pregnant women with mild/moderate anemia were examined first to find out the initial haemoglobin level and divided into desired number of groups. The experiment alone was given *Spinacia oleracea, solanum lycopersicum in* combination with honey and no such treatment was provided to the control group.	Herbal syrup of *Spinacia oleracea, Solanum lycopersicum* in combination with honey is a 5 times better treatment to enhance the hemoglobin level in pregnant women as compared to the group without the intervention of herbal syrup.	The combination of herbs with honey was beneficial for curing the disorder.	[56]

*Tinospora cordifolia* (Willd.), commonly known as Giloy, Guduchi, or Gulje, is classified within the Kingdom Plantae and the Division Magnoliophyta. It falls under the Order Ranunculales, the Family Menispermaceae, and the Genus *Tinospora*, with the specific species name being *cordifolia*. Giloy, Guduchi, or Gulje, is a significant plant in traditional medicine, known for its various therapeutic properties and applications. Its taxonomic classification highlights its association with the Menispermaceae family and the Ranunculales order, providing valuable insights into its botanical categorization within the broader context of plant taxonomy.

**Distribution:***Tinospora cordifolia* is endemic to Myanmar, Thailand, India, Sri Lanka, China, Philippines, Bangladesh, Malaysia, and South Africa. In India, it is distributed throughout the subtropical and tropical regions [57].

**Fig. 11:**
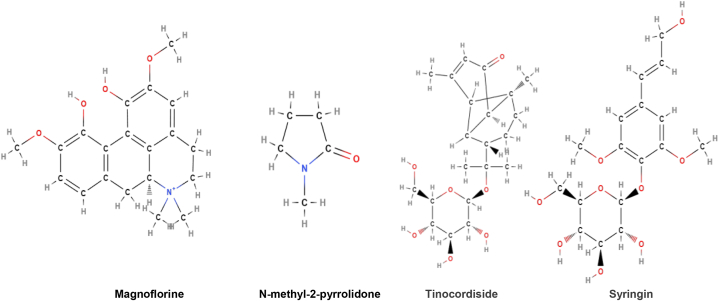
Active compounds of *Tinospora cordifolia*.

**Active constituents:** Various active compounds of the plant belong to diterpenoid lactones, aliphatic compounds, steroids, alkaloids, glycosides, polysaccharides, sesquiterpenoid, etc. It is having strong immunomodulatory property for which the active compounds like N-formylannonain, cordifolioside A, 11-hydroxymustakone, N-methyl-2-pyrrolidone, magnoflorine, tinocordiside and syringin have been reported to exhibit potential cytotoxic and immunomodulatory effects (Fig. 11) [58].

**Pharmacological** Effects**:** *Tinospora cordifolia* possesses medicinal properties such as immunomodulatory, antioxidant, antimicrobial, anti-toxic, antidiabetic, radio sensitizing, radioprotective, larvicidal, antipsychotic, hypolipidemic, antihyperglycemic, antiosteoporosis, antineoplastic and anticancer properties(Table 12) [58,59].

**Table 12:** Therapeutic potential of herbal honey preparation with *Tinospora cordifolia* against*diabetes mellitus*.

Model	Method	Intervention (Activity)	Outcome	Reference
Wistar strain albino rats	24 wistar albino rats were selected and divided into desired number of groups: Group 1: Treated with normal water and kept as control Group 2: Diabetic control group Group 3: Diabetic control + *Tinospora cordifolia* and honey suspension (42.34 mg/kg) Group 4: Diabetic control + Glibenclamide (0.45 mg/kg body wt.)	Rats were given streptozotocin for inducing diabetes (40 mg/kg body weight).After observing every5, 10, 15, and 20 days by one touch strip method, it was demonstrated that Group 3 (GG) has better potential in treatment of diabetes followed by (RS), (NC) and (DC).	It has been concluded from the overall experimental analysis that diabetes mellitus was better managed/controlled using herbal extract of Guduchi and honey.	[60]

## *13* *Trigonella foenum-graecum* L

*Trigonella foenum-graecum* L., commonly known as Fenugreek or Methi, is classified within the Kingdom Plantae and the Division Magnoliophyta. It is categorized under the Order Fabales, the Family Fabaceae, and the Genus *Trigonella*, with the specific species name being *foenum-graecum*. Fenugreek, or Methi, is a valuable plant known for its culinary and medicinal significance, with applications in various cuisines and traditional medicine systems. Its taxonomic classification underscores its association with the Fabaceae family and the Fabales order, providing valuable insights into its botanical placement within the broader context of plant taxonomy.

**Distribution:** It is an annual herb, having its origin in Central Asia. It is extensively disseminated throughout the world. *Trigonella foenum-graecum* is largely cultured in Argentina, India, China, Iran, Nepal, Pakistan, Afghanistan, Ethiopia, Greece, North Africa, Turkey, Egypt and Morocco [61].

**Fig. 12:**
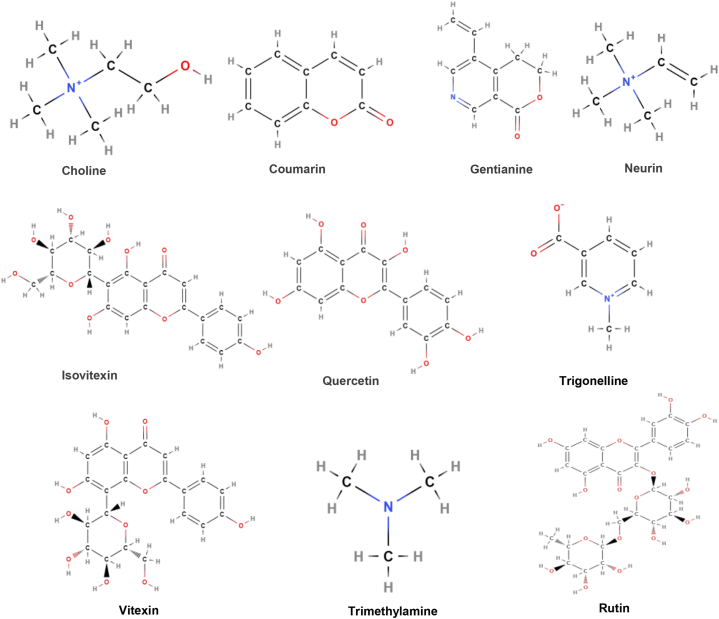
Active compounds of *Trigonella foenum-graecum*.

**Active constituents:***Trigonella foenum-graecum* contains active compounds like alkaloids, saponins, flavonoids and steroids (Snehlata and Payal, 2012). Saponins (Fenugrin B, Graecunins, Trigofoenosides A-G, Fenugreekine); Alkaloids (Trimethylamine, Choline, Neurin, Gentianine, Trigonelline, Betainand Carpaine); Amino acids (Isoleucine, 4-Hydroxyisoleucine, Leucine, Histidine, l-tryptophan, lysine, Arginine); Flavonoids (Rutin, Quercetin, Vitexin, Isovitexin); Steroidalsapinogens (Diosgenin, Yamogenin, Smilagenin, Tigogenin, Sarsasapogenin, Gitogenin, Neotigogenin, Neogitogenin, Saponaretin, Yuccagenin); Fibers (Gum, Neutral Detergent Fiber); Lipids (Monoacylglycerols, Diacylglycerols,Triacylglycerols, Phosphatidylcholine, Phosphatidylinositol, Phosphatidylethanolamine, Free Fatty Acids); Coumarin; Minerals; Vitamins; 22%Proteins and 28%Mucilage(Fig. 12) [62].

**Pharmacological Effects:** Fenugreek possesses antibacterial, antidiabetic, antioxidant, anticancer, anti-inflammatory, antitumor, hypocholesterolaemic, immunomodulatory and hypoglycaemic activities (Table 13) [62,63].

**Table 13:** Therapeutic potential of *Trigonella foenum-graecum* combined with honey used for promoting breastfeeding practice.

Model	Method	Intervention (Activity)	Outcome	Reference
Breast- feeding mothers	75 potential breast feeding patients were selected: 1. Fenugreek and Honey group: It included 36 women 2. Control (Fenugreek) group: It included 39 women	Fenugreek when fortified with honey produced significant effect on breastfeeding success (BFS) in comparison to those provided fenugreek alone.	Fortification of fenugreek with honey produces natural galactagogue which is required for enhancing success of breastfeeding.	[64]

## *14* *Zingiber officinale*Roscoe

*Zingiber officinale* Roscoe, commonly known as Ginger or Adrak, belongs to the Kingdom Plantae and the Division Magnoliophyta. It is classified under the Order Zingiberales, the Family Zingiberaceae, and the Genus *Zingiber*, with the specific species name being *officinale*. Ginger, or Adrak, is a widely recognized and extensively used spice and medicinal plant, celebrated for its distinctive flavor and various health benefits. Its taxonomic classification highlights its affiliation with the Zingiberaceae family and the Zingiberales order, emphasizing its position within the broader context of plant taxonomy.

**Distribution:** The plant is native to Indo-Malayan region; India and China are considered to be the centre of origin of ginger. It is widely disseminated in Asia, Australia, Africa and America [65].

**Fig. 13:**
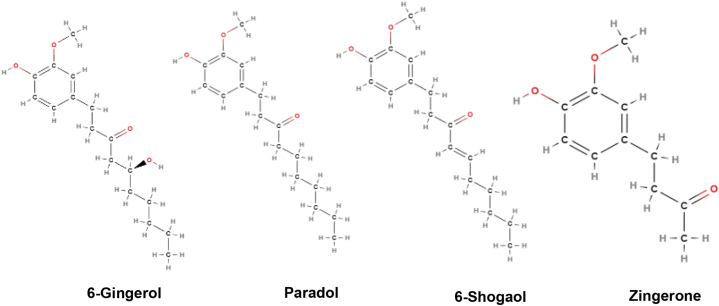
Active compounds of *Zingiber officinale*.

**Active constituents:** The active components of *Zingiber officinale* include gingerols, shogaols, paradols, 3-dihydroshogaols, dihydroparadols, acetyl derivatives of gingerols, gingerdiols, mono- and di-acetyl derivatives of gingerdiols, 1- dehydrogingerdiones, diarylheptanoids, and methyl ether derivatives of some of these compounds. Moreover [6],-gingerol (1) [4],- [7],- [8],-, and [10]-gingerol (3–6), methyl [4]-gingerol, methyl [8]- gingerol [4].- [6],- [8],- [10],- and [12]-shogaol, methyl [4]-, methyl [6]- and methyl [8]-shogaol were identified. Paradols are 5-deoxygingerols [6].-Paradol (11), along with [7]- [8],- [9],- [10],- [11],-, and [13]- paradols were characterized as methyl [6]-paradol (Fig. 13) [66].

**Pharmacological Effects:***Zingiber officinale* shows hypocholesterolemic, hypoglycemic, and hypolipidemic properties. It also possesses anthelmintic, anti-inflammatory, analgesic, antiviral, anti-fungal and anticancer properties (Table 14) [66].

**Table 14:** The activity of a combination of ginger, onion*,* lemon and honey in rats maintained at high cholesterol diet.

Model	Method	Intervention (Activity)	Outcome	Reference
Sprague Dawley (SD) rats	36 rats were selected and divided in desired number of groups.Group I was given normal chow diet-fed (NC), Group II was given high cholesterol diet (HCD), Group III was given HCD + Simvastatin, and the rest three groups were given HCD + ZACAH extracts in varying doses (1, 3 and 5 mg/kg body weight) for 18 weeks. Simvastatin was used as control.	Administration of ZACAH extracts reduced the level of total cholesterol (TC), low-density lipoprotein (LDL), triglycerides (TG), and amplified the level of HDL.It exhibited therapeutic effects in rats fed with HCD.	It has been concluded that ZACAH extracts enhanced hyperlipidaemia inrats. Therefore, ZACAH extracts can be employedto treat hyperlipidaemia.	[67]

## 15 Summary

The combination of honey and herbs has been esteemed for its therapeutic potential in managing various pathological diseases. This comprehensive review delves into the intricate mechanisms that underlie the synergistic effects of honey and herbs, shedding light on their combined efficacy in combating a spectrum of ailments. Furthermore, honey infused with herbs is significant as a promising avenue for the development of novel therapeutic strategies, emphasizing its potential as a holistic approach to disease management. Notably, the historical integration of honey and herbs in traditional medicine has fueled recent scientific investigations, uncovering a myriad of bioactive compounds present in both components.

Phenolic compounds, flavonoids, and essential oils found in honey and medicinal herbs collectively contribute to the therapeutic properties of the infused mixture [73. The critical analysis of the molecular mechanisms underlying the efficacy of honey infused with herbs in managing pathological diseases reveals multifaceted pathways, including antioxidant, anti-inflammatory, immune modulation, and antimicrobial activities [68,69]. These insights provide compelling evidence for its potential in mitigating various ailments.

Therapeutic applications of honey infused with herbs might be used for the treatment of specific pathological conditions, such as respiratory disorders, gastrointestinal ailments, dermatological issues, and metabolic syndromes. It has been evidenced and supported by previous studies and traditional uses of honey and herbs, which may support the idea that honey infused with herbs could be a promising therapeutic intervention in pathological disease management.

However, to bring this therapy up to the level of implementation, focus has been given to advanced extraction techniques and formulation optimization. The integration of honey-infused herbal preparations into mainstream healthcare holds promise for novel and holistic therapeutic interventions. By leveraging the synergistic potential of honey and herbs, it could set the stage for a holistic 0approach to disease management, ultimately aiming to alleviate the burden of pathological ailments on global health.

## 16 Conclusion

Herbal therapy is the best treatment for a healthy and disease-free life. It is a well-known fact that herbal remedies are better than conventional treatments for treating diseases and do not have side effects. A combination of honey and herbs is the best home remedy for healing wounds, preventing asthma symptoms, improving anaemia in pregnant women, boosting immunity, and living a healthy life. In the present article, 12 species of plants have been selected from 10 families, each of which has proven to have its own therapeutic properties when combined with honey for the treatment of various ailments. There are three species in the family *Zingiberaceae, i.e., Curcuma xanthorriza Roxb., Zingiber officinale Roscoe, and Alpinia galanga* L., whose antioxidant, cytotoxic, immunomodulatory, anti-hyperlipidemic, and anti-asthmatic properties have been well demonstrated in experiments. Plant species like *Nigella sativa* L., *Allium sativum* L., *and Sesamum indicum* L. have been experimentally proven to exhibit wound-healing properties in rats. The plant *Curcuma xanthorriza* Roxb. shows antioxidant and immunomodulatory activities against carcinogenesis. *Tinospora cordifoliais* is reported to work well against diabetes. *Capparis spinosa* improves the toxic effects of trichloroacetic acid. Euphorbia hirtais is used to cure stomach ulcers in rats. *Trigonella foenum-graecum* may be used to modulate breastfeeding in pregnant women. *Phyllanthus emblica* shows its therapeutic potential for the treatment of gastroesophageal reflux diseases in rats. *Spinacia oleracea* is used for enhancing haemoglobin levels in pregnant women. All these plants combined with honey showed significant effects in treating various types of diseases. It has been concluded in this study that herbal formulations (herbs combined with honey) have manyfold higher effectiveness in the treatment of diseases as compared to herbs alone or modern medicinal systems.

## 17 Novelty

Pathological diseases, ranging from infectious to chronic conditions, have a profound impact on global health. Conventional treatments often face limitations, such as antimicrobial resistance and adverse effects, emphasizing the need for novel therapeutic options. Honey, renowned for its medicinal properties, exhibits antimicrobial, anti-inflammatory, and wound-healing activities. Combining honey with various herbs can potentially enhance these therapeutic properties and offer novel approaches to disease management.

**Honey-Herb Infusions:** The unique chemical composition of honey, enriched with various vitamins, minerals, and enzymes, serves as an excellent medium for herbal infusion. Herbs like ginger, turmeric, garlic, and thyme are among the widely studied candidates for their synergistic effects when combined with honey. This section explores the potential bioactive interactions that occur during the infusion process and their contribution to the overall therapeutic benefits.

**Antimicrobial Potential:** Pathogens' growing resistance to conventional antibiotics has spurred the search for alternative antimicrobial agents. The review delves into the antimicrobial efficacy of honey-herb infusions against a broad spectrum of bacteria, fungi, and even some viruses. The mechanisms of action, including disruption of microbial cell membranes and inhibition of microbial enzymes, are discussed in detail.

**Anti-inflammatory Properties:** Chronic inflammation underpins numerous pathological diseases, necessitating anti-inflammatory interventions. The anti-inflammatory potential of honey-herb infusions is examined, elucidating the modulatory effects on key inflammatory mediators.

**Wound Healing:** Honey has long been utilized for wound care due to its wound-healing and tissue regeneration properties. This section evaluates how the combination with specific herbs further enhances these effects, promoting tissue repair, and accelerating wound closure.

**Immunomodulatory Effects:** A sound immune system is crucial for combating infections and maintaining overall health. The review explores the immunomodulatory activities of honey-herb infusions, focusing on their ability to bolster the immune response against pathogens while preventing excessive inflammation.

**Clinical Applications:** To validate the therapeutic potential of honey-herb infusions, evidence from preclinical studies and clinical trials is critically analyzed. Promising results from various pathological conditions, including respiratory infections, gastrointestinal disorders, and skin ailments, are highlighted, supporting the plausibility of these infusions as adjunctive therapies.

**Safety Considerations:** The safety profile of honey-herb infusions is paramount for their clinical application. Potential adverse effects, interactions with medications, and allergic reactions are assessed to provide a comprehensive understanding of their safety and limitations.

## 18 Recommendations

**Research Advancement:** Invest in further research to uncover the specific bioactive compounds and molecular mechanisms behind the therapeutic effects of honey-infused herbs, which will strengthen the scientific basis for these treatments.

**Clinical Trials:** Conduct large-scale, well-designed clinical trials to validate the efficacy and safety of honey-infused herb therapies across various pathological diseases, ensuring robust evidence for clinical application.

**Combination Optimization:** Explore and optimize the combinations of herbs with different types of honey to maximize their synergistic effects and therapeutic potential.

**Antimicrobial Resistance:** Investigate honey-infused herb therapies as potential alternatives to conventional antibiotics in addressing antimicrobial resistance challenges.

**Integrative Medicine Collaboration:** Encourage collaboration between traditional medicine practitioners and modern healthcare providers to integrate honey-infused herb therapies into mainstream healthcare systems.

**Personalized Medicine Application:** Explore personalized medicine approaches to tailor honey-infused herb treatments based on individual patient profiles, optimizing treatment outcomes.

**Safety Monitoring:** Continuously monitor and assess the safety profile of honey-infused herb therapies, particularly in combination with other medications.

**Public Awareness**: Increase awareness among healthcare professionals and the general public about the potential benefits of honey-infused herb therapies for various pathological diseases.

**Regulatory Consideration:** Engage regulatory agencies to consider official approval or guidelines for the use of honey-infused herb therapies in disease management.

**Sustainable Sourcing:** Ensure sustainable sourcing of honey and herbs to support long-term availability and environmental conservation.

## 19 Future prospective

The review highlights exciting future prospects for honey-infused herb therapies. Anticipated advancements include advanced formulations, mechanistic insights, rigorous clinical trials, combatting antimicrobial resistance, novel delivery systems, integration into global medicine, personalized treatments, regulatory approvals, and exploration of uncharted herbs. Together, these developments may revolutionize pathological disease management and promote innovative therapeutic solutions.

## 20 Ethics approval and consent to participate

Not applicable.

## 21 Consent for publication

All authors approved the final form of the manuscript and agreed to its publication.

## Funding

It is declared that no funding has been received to design this manuscript from any agency/institution.

## Data availability statement

Manuscript doesn't does not contain any data.

## Declarations consent to participate

All listed authors have been approved to participate in the manuscript.

## CRediT authorship contribution statement

**Suresh Kumar:** Writing – original draft. **Mamta Verma:** Writing – original draft. **Younis Ahmad Hajam:** Writing – review & editing. **Rajesh Kumar:** Investigation, Conceptualization.

## Declaration of competing interest

The authors declare the following financial interests/personal relationships which may be considered as potential competing interests.
